# Unilateral biportal endoscopic discectomy combined with annulus fibrosus suturing for lumbar disc herniation: a retrospective study of early clinical and radiological outcomes

**DOI:** 10.3389/fsurg.2026.1836793

**Published:** 2026-05-22

**Authors:** Jiale Liu, Yan Sun, Jifu Sun

**Affiliations:** Department of Orthopaedics, Affiliated Hospital of Jiangsu University, Zhenjiang, Jiangsu, China

**Keywords:** annulus fibrosus suturing, discectomy, lumbar disc herniation, recurrence, unilateral biportal endoscopy

## Abstract

**Objective:**

To evaluate the clinical efficacy and radiological outcomes of unilateral biportal endoscopic (UBE) discectomy combined with annulus fibrosus suturing compared with UBE discectomy alone in the treatment of lumbar disc herniation (LDH).

**Methods:**

A total of 62 LDH patients with LDH who underwent UBE surgery at the Affiliated Hospital of Jiangsu University between February and June 2024 were enrolled. Patients were divided into two groups: the discectomy with annulus suture group (Suture Group, *n* = 30) and the discectomy-alone group (Discectomy Group, *n* = 32). General patient data, operative time, time to first ambulation, and hospital stay were recorded. The Visual Analog Scale (VAS) was used to evaluate pain in the lower back and legs, while the Oswestry Disability Index (ODI) assessed functional status, both preoperatively and at multiple postoperative follow-up visits. Intervertebral disc height and Pfirrmann grade were evaluated preoperatively and at 1 year postoperatively to assess degenerative changes. Recurrence rates at 1 year and clinical outcomes (modified MacNab criteria) were analyzed.

**Results:**

All operations were completed successfully, and all patients were followed up for more than 1 year. There were no statistically significant differences between the two groups in postoperative ambulation time or length of hospital stay (*P* > 0.05). However, the operative time was significantly longer in the Suture Group than in the Discectomy Group (*P* < 0.05). At all postoperative time points, both the VAS scores for low back and leg pain and the ODI were significantly improved compared with preoperative values in both groups (*P* < 0.05). The leg pain VAS score at 3 days postoperatively was significantly lower in the Suture Group than in the Discectomy Group, whereas no statistically significant between-group differences (*P* > 0.05). At the final follow-up, the intervertebral disc height at the operated level decreased by 8.98% compared with the preoperative value in the annular Suture Group and by 10.09% in the Discectomy Group, with no statistically significant difference between the two groups (*P* > 0.05). Progression of disc degeneration was observed in two patients in the Discectomy Group at the final follow-up, whereas no aggravation of disc degeneration was noted in the Suture Group. Two cases of same-level postoperative recurrence occurred in the Discectomy Group, corresponding to a recurrence rate of 6.25%, whereas no recurrence was observed in the Suture Group. According to the modified MacNab criteria at the final follow-up, there was no statistically significant difference in the excellent-to-good rate between the two groups (*P* > 0.05). In the Suture Group, two patients experienced transient aggravation of postoperative lower extremity numbness, which improved before discharge after conservative treatment including dehydration therapy and corticosteroids.

**Conclusion:**

In selected patients with single-level lower lumbar paracentral LDH, both UBE discectomy alone and UBE combined with annular suturing provided favorable short-term outcomes.

## 1 Introduction

Lumbar disc herniation (LDH) is one of the most common disorders encountered in spinal surgery. Herniated nucleus pulposus compresses the nerve roots, leading to low back pain, radicular leg pain, and neurological dysfunction in the lower extremities. Patients who do not respond to conservative treatment often require surgical intervention (1). Surgical approaches primarily include open procedures and a variety of minimally invasive techniques. Minimally invasive discectomy is currently recognized as an effective and less invasive treatment for LDH (2). However, long-term follow-up studies after discectomy have reported a same-level recurrent disc herniation rate of 10%–15% (3). Some scholars have suggested that the original annular defect and iatrogenic tears created during surgery may be major contributors to persistent low back and leg pain, accelerated disc degeneration, disc space narrowing, and even postoperative recurrence (4). At present, biological repair strategies for the annulus fibrosus, such as tissue engineering reconstruction and gene editing, remain largely experimental, whereas surgical repair techniques have been partially implemented in clinical practice (5). The efficacy of annular repair has been documented in existing literature. For instance, Yao Dongyuan et al. (6) performed nucleotomy combined with annular suturing using an expandable tubular retractor system and found that suturing effectively reduced the risk of postoperative recurrence and reoperation. In a comparative study, He Bo et al. (7) reported that patients undergoing endoscopic transforaminal nucleotomy combined with annular suture (*n* = 208) had significantly lower rates of recurrence and reoperation compared to those receiving nucleotomy alone (*n* = 204), with a statistically significant difference between the groups. However, in these procedures, the quality and reproducibility of the suturing are often questioned due to poor visualization caused by bleeding on the annular surface in an air medium, or excessively narrow working channels and limited space. For example, Jiang Guoqiang et al. (8) reported a failure rate of 17.5% (7/40) for annular suturing performed under endoscopic transforaminal surgery.Unilateral Biportal Endoscopy (UBE), a rapidly evolving endoscopic spinal technique in recent years, has been widely applied in the treatment of degenerative spinal conditions, including lumbar disc herniation, owing to its clear visualization and extensive operative field (9). Nevertheless, there are few reports on annular suturing under UBE, and existing studies lack comprehensive evaluation of imaging indicators. Therefore, this study aimd to compare the early clinical and radiological outcomes of UBE-assisted discectomy combined with annular suturing vs. UBE-assisted discectomy alone in the treatment of lumbar disc herniation.

## 2 Materials and methods

### 2.1 Patient selection criteria

Inclusion criteria: (1) Patients with lumbar disc herniation (LDH) consistent in clinical symptoms, signs, and imaging findings, and ineffective after conservative treatment; (2) Treated with either unilateral biportal endoscopic (UBE) discectomy combined with annulus fibrosus suturing or UBE discectomy alone, with a relatively preserved annular integrity after disc removal; (3) Imaging findings suggesting soft disc herniation without significant calcification; (4) Pfirrmann grade III or IV discs; (5) Imaging-based classification of disc herniation located in the paramedian zone; (6) Follow-up longer than 12 months with complete follow-up data.

Exclusion criteria: (1) Combined lumbar instability, lumbar spinal stenosis, etc; (2) Concurrent spinal infections, spinal deformities, or tumors; (3) History of previous lumbar surgery; (4) Long-term use of anticoagulants, coagulation disorders, or other conditions potentially increasing intraoperative bleeding risk; (5) Patients with neuropsychiatric disorders unable to accurately complete relevant scales.

All procedures were performed after obtaining informed consent from the patients and by the same spinal surgery team. This study was approved by the Ethics Committee of Jiangsu University.

### 2.2 General data

A total of 62 patients were enrolled in this study. Based on the surgical approach, they were divided into two groups: the Discectomy Group (UBE discectomy alone, 32 cases) and the Suture Group (UBE discectomy combined with annular suturing, 30 cases). Baseline characteristics are shown in Table 1. There were no statistically significant differences between the two groups in age, gender, body mass index (BMI), duration of disease, or responsible segments between the two groups (*P* > 0.05).

**Table 1:** Baseline characteristics of the two groups.

Variables	Discectomy group (*n* = 32)	Suture group (*n* = 30)	*P*
Age (year, $\bar{x}\pm s$)	48.69 ± 16.77	46.5 ± 15.44	0.596
Gender (*n*, male/female)	22/10	18/12	0.472
BMI (kg/m^2^, $\bar{x}\pm s$)	26.29 ± 2.53	25.22 ± 1.73	0.058
Course of disease (months, $\bar{x}\pm s$*s*)	4.36 ± 0.49	4.46 ± 0.37	0.406
Responsible levels (L3-4/L4-5/L5-S1)	5/13/14	4/12/14	0.788

### 2.3 Surgical approach

**Figure 1:**
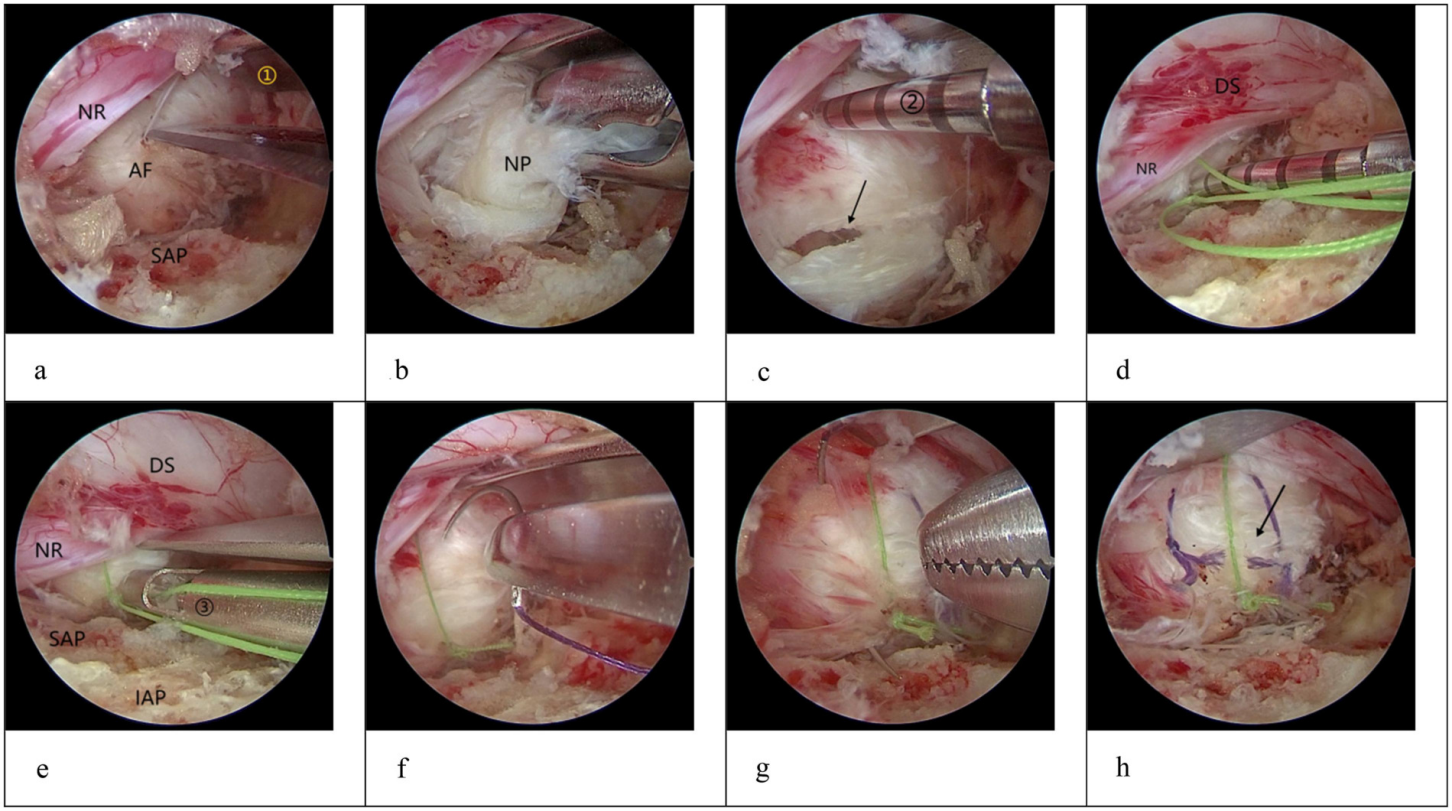
Intraoperative procedure [NR, nerve root; AF, Annulus fibrosus; SAP, superior articular process, IAP, inferior articular process; NP, nucleus pulposus; DS, dural sac, black arrow: annular fissure, (1): nerve root retractor, (2): disposable annulus fibrosus closure device, (3), integrated knot pusher and suture cutter). **(a)** Intraoperatively, a contained disc herniation was observed. An incision was made at the weakest point of the annulus fibrosus.; **(b)** The nucleus pulposus was removed with nucleus pulposus forceps; **(c)** The first suture was passed from the inner side of the annular fissure using the disposable annular closure device; **(d)** The second suture was passed from the outer side of the annular fissure using the disposable annular closure device; **(e)**:The knot was advanced and tightened, and the suture was cut; **(f)**:The first suture was placed manually using 4-0 absorbable suture; **(g)** The second suture was placed manually using 4-0 absorbable suture; **(h)**:The closure was completed. Three parallel sutures were observed, with the annular fissure tightly closed and a smooth surface.

(1) Discectomy Group: Patients underwent general anesthesia and were placed in a prone position with the abdomen free. Using C-arm fluoroscopy, the operating table was adjusted under anteroposterior view until the target intervertebral space was perpendicular to the ground. The surface projections of the pedicles at the target level were marked. A transverse line was drawn at the junction of the spinous process and lamina, and a longitudinal line was drawn along the medial borders of the upper and lower pedicles. The intersection points of these lines were identified, and incisions were made 1.5 cm proximal and distal to these points. After sterilization and draping, the target level was reconfirmed using C-Arm x-Ray machine. A working channel was established, and the endoscope system and light source were connected. Soft tissues in the interlaminar space were cleared using a radiofrequency knife to expose the lower edge of the upper lamina and the upper edge of the lower lamina. Part of the lower edge of the upper lamina and the upper edge of the lower lamina were removed using laminectomy rongeurs, bone drills, and nucleus forceps until the proximal and distal attachments of the ligamentum flavum were visualized. The ligamentum flavum was preserved and lifted to expose the dura mater and nerve root. After achieving hemostasis, the nerve root and dural sac were retracted medially from the lateral edge of the nerve root to expose the protruding disc tissue. For non-contained herniations, the herniated fragments were removed through the rupture site. For contained herniations, an appropriate incision was made in the annulus fibrosus. The herniated nucleus pulposus and loose intradiscal fragments were removed using nucleus forceps. The annular rupture was coagulated using a radiofrequency electrode, and hemostasis in the spinal canal was achieved. The canal was re-explored to rule out hidden compression, and after confirming adequate decompression, a drainage tube was placed. The working channel was withdrawn, and the incision was sutured.

(2) Suture Group: Nucleus pulposus removal was performed in the same manner as in the control group. After excision of the herniated nucleus pulposus, a nerve root retractor was used to shield the dura mater and the nerve root, with the assistant maintaining stable retraction. The surgeon first inserted a non-looped suture needle (No. 2 suturing device) and punctured the annulus fibrosus at a point 3–4 mm from the margin of the annular defect on the side adjacent to the nerve root. After penetrating the annulus fibrosus, the trigger was activated to deploy the suture anchor. Once correct placement was confirmed, the needle was withdrawn slowly. Next, the looped suture needle (No. 1 suturing device) was prepared extracorporeally by pre-threading its loop through the suture delivered by the No. 2 device. The No. 1 needle was then introduced through the annulus fibrosus at the corresponding entry point on the contralateral side of the defect, mirroring the insertion site of the No. 2 device. After penetration of the annulus fibrosus, the trigger was activated to complete deployment. Upon confirmation of appropriate placement, the needle was gradually withdrawn. Maintaining appropriate tension on the No. 2 suture, the No. 1 suture was pulled to approximate and tighten the annular defect. The knot was secured and advanced away from the nerve root side, followed by two additional sequential knot advancements to ensure firm fixation. Depending on the degree of defect closure, one to two supplementary stitches were placed using 4-0 absorbable sutures for reinforcement. After completion of the repair, satisfactory closure of the annular defect was confirmed and excess suture material was trimmed. Hemostasis within the spinal canal was achieved using a radiofrequency electrode. The spinal canal was re-explored to rule out occult compression. After confirming adequate decompression, a drainage tube was placed and the working channel was withdrawn. The incision was then closed in layers and dressed in a sterile manner. The representative intraoperative procedure is shown in Figure 1.

### 2.4 Postoperative management and efficacy evaluation metrics

Routine postoperative analgesia and dehydration therapy were implemented. The drainage tube was removed 24 h after surgery. Bed-based functional exercises were initiated on the first postoperative day. Patients began ambulatory activities with a lumbar brace on the second postoperative day. Strict use of the lumbar brace was maintained for 6 weeks, during which back muscle functional exercises were gradually intensified. Regular outpatient reviews were scheduled at 6 weeks, 6 months, and 12 months postoperatively.

**Figure 2:**
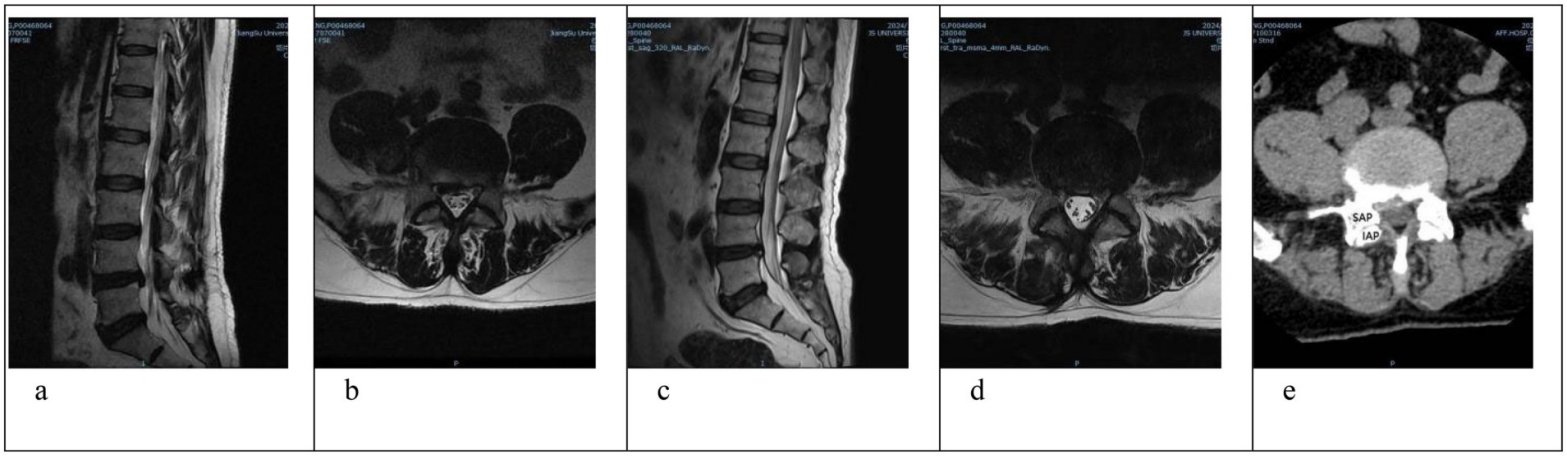
A 59-year-old male patient. **(a)** Preoperative sagittal lumbar MRI showing an L4/5 disc herniation. **(b)** Preoperative axial lumbar MRI shows a right paracentral disc herniation compressing the nerve root and dural sac. **(c,d)** One-year follow-up lumbar MRI shows complete removal of the herniated nucleus pulposus following UBE-assisted discectomy combined with annular suturing, adequate nerve root decompression, no recurrence of disc herniation, and no progression of disc degeneration. **(e)** Postoperative axial lumbar CT shows complete removal of the herniated nucleus pulposus and well-preserved facet joints.

Operative time, perioperative complications, time to ambulation, and length of hospital stay were recorded for both groups. Data on low back pain and leg pain visual analog scale (VAS) scores and Oswestry Disability Index (ODI) were collected preoperatively and at 3 days, 6 weeks, 6 months, and 12 months postoperatively. The intervertebral disc height of the affected segment was measured preoperatively and at the final follow-up. The intervertebral disc height was defined as the distance between the midpoint of the line connecting the lower edges of the superior vertebral body and the midpoint of the line connecting the upper edges of the inferior vertebral body at the diseased segment. This was measured three times by the same orthopedic surgeon, and the average value was taken. The degree of disc degeneration according to the Pfirrmann grading system on MRI was recorded for both groups preoperatively and at the final follow-up. Assessments were performed independently by two experienced spine surgeons. In cases of disagreement between the two reviewers, consensus was reached through discussion; if necessary, a third senior physician made the final determination. All radiological assessments were blinded to the patients’ clinical information, group allocation, and follow-up outcomes. Representative imaging findings are shown in Figure 2. Therapeutic efficacy was evaluated using the modified MacNab criteria at 12 months postoperatively.

### 2.5 Statistical analysis

All statistical analyses were performed using SPSS version 30.0. Continuous variables conforming to a normal distribution were expressed as mean ± standard deviation ($\bar{x}\pm s$). Intergroup comparisons were conducted using the independent-samples *t*-test, while preoperative and postoperative comparisons within the same group were analyzed using the paired-samples *t*-test. Categorical variables were compared between groups using the chi-square test. Ordinal variables were analyzed using the Mann–Whitney *U* test for between-group comparisons and the Friedman test for repeated within-group comparisons (e.g., Pfirrmann grade). A two-sided *P* value < 0.05 was considered statistically significant.

## 3 Results

### 3.1 Perioperative data

All 62 patients with LDH successfully underwent surgery. Perioperative data are summarized in Table 2. There were no statistically significant differences between the two groups in postoperative time to ambulation or length of hospital stay after surgery (*P* > 0.05). The operative time in the suture group was significantly longer than that in the Discectomy group (*P* < 0.05). No intraoperative complications, such as nerve injury or dural tears, occurred in either group. Postoperatively, no epidural hematoma, infection, or other related complications were observed. In the Suture group, two patients experienced transient aggravation of lower limb numbness after surgery. Symptoms improved before discharge following conservative management, including dehydration therapy, corticosteroids, and neurotrophic treatment.

**Table 2:** Comparison of perioperative data between the two groups.

Variables	Discectomy group **(***n* = 32**)**	Suture group **(***n* = 30**)**	*P*
Operative time (min, $\bar{x}\pm s$**)**	41.94 ± 3.56	76.39 ± 9.61	<0.001
Postoperative ambulation time (d, $\bar{x}\pm s$**)**	2.1 ± 0.32	2 ± 0.26	0.216
Hospitalization days (d, $\bar{x}\pm s$**)**	8.84 ± 1.73	9.23 ± 1.85	0.394
Complications [*n*]	0	2	0.230

### 3.2 Follow-up results

All patients were followed up for more than 12 months. Follow-up outcomes are shown in Table 3. In both groups, VAS scores for low back pain and leg pain and ODI scores at all postoperative time points were significantly improved compared with preoperative values, with statistically significant differences (*P* < 0.05). At postoperative day 3, the leg pain VAS score in the Suture group was significantly lower than that in the Discectomy group (*P* < 0.05), whereas no significant between-group differences were observed at other postoperative time points (*P* > 0.05). Radiological outcomes are summarized in Table 4.

**Table 3:** Comparison of follow-up outcomes between the two groups (x ± s).

Variables	Discectomy group (*n* = 32)	Suture group (*n* = 30)	*P*
Low back pain VAS			
Preoperative	5.73 ± 1.16	5.49 ± 0.51	0.290
3 days after surgery	3.14 ± 0.32	3.03 ± 0.16	0.104
6 weeks after surgery	2.18 ± 0.36	2.12 ± 0.17	0.476
6 month after surgery	2.05 ± 0.32	1.95 ± 0.19	0.132
Final follow-up	1.59 ± 0.37	1.52 ± 0.17	0.413
*P*	<0.05	<0.05	
Leg pain VAS			
Preoperative	7.23 ± 1.01	7.07 ± 0.99	0.514
3 days after surgery	4.71 ± 0.25	3.21 ± 0.37	<0.001
6 weeks after surgery	2.24 ± 0.23	2.17 ± 0.21	0.201
6 month after surgery	1.61 ± 0.23	1.56 ± 0.15	0.320
Final follow-up	1.25 ± 0.30	1.16 ± 0.12	0.101
*P*	<0.05	<0.05	
ODI **(**%**)**			
Preoperative	68.73 ± 1.87	69.31 ± 2.33	0.287
3 days after surgery	49.49 ± 2.20	48.89 ± 2.41	0.308
6 weeks after surgery	33.22 ± 1.75	32.88 ± 1.58	0.424
6 month after surgery	24.59 ± 1.24	24.23 ± 1.01	0.217
Final follow-up	14.43 ± 0.84	14.61 ± 0.84	0.383
*P*	<0.05	<0.05	
Recurrence (%)	6.25%	0	0.492

**Table 4:** Comparison of radiological outcomes between the two groups.

Variables	Discectomy group (*n* = 32)	Suture group (*n* = 30)	*P*
Intervertebral space height (mm, *x* ± *s*)			
Preoperative	11.55 ± 1.27	11.59 ± 1.32	0.896
Final follow-up	10.25 ± 0.44	10.58 ± 1.47	0.229
*P*	<0.001	0.005	
Pfirrmann Grading (Ⅱ/Ⅲ/Ⅳ/Ⅴ)			
Preoperative	0/10/22/0	0/11/19/0	0.661
Final follow-up	0/8/24/0	0/11/19/0	0.328
*P*	0.587	1	

There was no significant difference in preoperative intervertebral disc height between the two groups. At the final follow-up, the intervertebral disc height at the operated level had decreased by 10.09% in the Discectomy group and by 8.98% in the Suture group compared with the preoperative measurements. The reduction in disc height was statistically significant within each group compared with baseline; however, the between-group difference was not statistically significant (*P* > 0.05).

Lumbar magnetic resonance imaging (MRI) was performed at the final follow-up. According to the Pfirrmann classification, two patients in the Discectomy group progressed from grade III to grade IV at 1 year postoperatively, whereas no progression of disc degeneration was observed in the Suture group. There was no statistically significant difference between the two groups.

At the final follow-up, clinical outcomes assessed using the Modified Macnab criteria were as follows: in the Discectomy group, 24 cases were rated as excellent, 5 as good, 1 as fair, and 2 as poor; in the Suture group, 25 cases were rated as excellent, 4 as good, and 1 as fair. There was no statistically significant difference in the distribution of clinical outcomes between the two groups.

In the Discectomy group, two patients (6.25%) experienced recurrence at the same surgical level. One patient underwent unilateral biportal endoscopic (UBE) discectomy, and the other underwent UBE-assisted posterior lumbar fusion. No recurrence or reoperation occurred in the Suture group. Although recurrence occurred only in the Discectomy group, the difference between groups was not statistically significant (*P* = 0.492).

## 4 Discussion

The primary goal of discectomy for lumbar disc herniation (LDH) is neural decompression to relieve symptoms and preserve neurological function. Once adequate decompression has been achieved, additional efforts should be made to minimize damage to spinal stability and reduce the risk of recurrence. With the evolution of minimally invasive techniques and the emergence of the concept of targeted discectomy, current surgical strategies emphasize removal of only the herniated and loose nucleus pulposus tissue while preserving relatively healthy nucleus pulposus. However, residual nucleus pulposus has become an important factor contributing to postoperative recurrence (4).

Moreover, the intervertebral disc is a relatively enclosed and avascular structure with extremely limited intrinsic healing capacity. Scar repair of annular defects may require several weeks or even more than 1 year (10). Studies have shown that early recurrent herniation, particularly within 6 months after surgery, frequently occurs at the site of the surgically created annular defect (11). Previous investigations suggest that precise anatomical reduction allows close apposition of the outer annulus fibrosus, thereby creating favorable conditions for fibrous tissue ingrowth and scar remodeling (12). Annulus fibrosus suturing facilitates primary closure of the annular defect and may therefore promote more rapid repair while reducing the risk of early recurrence after discectomy.

In a study by Bailey et al. (3), 500 patients underwent annular closure with the Xclose device during open discectomy. Although no statistically significant difference was observed compared with the non-suture group, annular closure preserved the benefits of simple discectomy without increasing surgical risk and reduced, to some extent, the need for reoperation due to reherniation. Zhu Zhaoyin et al. (13) first reported annular repair following microendoscopic discectomy (MED), demonstrating that annular suturing effectively reduced postoperative recurrence. Han Kang et al. (14) subsequently reported annular repair under unilateral biportal endoscopy (UBE) for lumbar disc herniation, achieving favorable clinical outcomes, particularly in cases of giant herniation. In the present study, no recurrence was observed within one year after UBE-assisted discectomy combined with annular suturing, which is consistent with the findings of Bailey et al.

In addition, primary closure of the annular defect may reduce local scar formation, better restore the natural anatomical environment of the nerve root and dural sac, and alleviate neural adhesions. Annular suturing may also help prevent the leakage of inflammatory mediators from the intervertebral disc (15). Chen Wang et al. (16) compared perioperative serum and drainage fluid inflammatory cytokine concentrations between patients with and without annular suturing and found that discectomy combined with annular repair reduced inflammatory mediator exudation and release. This may attenuate inflammatory reactions in adjacent tissues and nerve roots and contribute to improved early postoperative neurological recovery. In the present study, the Suture group demonstrated lower leg pain VAS scores at postoperative day 3 compared with the Discectomy group, which may be attributable to this mechanism.

The degree of intervertebral disc degeneration directly affects physiological disc function. Disruption of annular integrity and loss of intradiscal pressure are major contributors to accelerated postoperative disc degeneration (17). Annular suturing may partially restore annular integrity and maintain intradiscal pressure, thereby delaying degenerative progression (18). Song Tongqu et al. (19) reported partial rehydration of the nucleus pulposus and reduced degeneration grades during follow-up in adolescent LDH patients treated with annular suturing, which was beneficial for maintaining lumbar function. In the present study, patients with Pfirrmann classification grade III–IV degeneration were included to evaluate postoperative degenerative progression at one year. Two patients in the Discectomy group progressed from grade III to grade IV, whereas no progression was observed in the suture group. Although intervertebral disc height decreased in both groups at one year compared with baseline, no significant intergroup difference was detected.

Compared with conventional fixed-channel endoscopic systems and transforaminal endoscopy, the unilateral biportal endoscopic (UBE) system features independent viewing and working channels, providing a wider surgical field and greater operative flexibility. In most cases, standard open surgical instruments can be used. Continuous saline irrigation reduces bleeding and improves visualization. These technical advantages theoretically facilitate annular suturing. However, annulus fibrosus suturing remains technically demanding and is associated with a relatively steep learning curve. The use of a single-use annular suturing device may help reduce technical complexity, whereas manual suturing should be performed only after sufficient proficiency has been achieved.

In this study, UBE-assisted discectomy was performed via a posterior interlaminar approach. For paracentral herniations, the surgical trajectory is perpendicular to the annulus fibrosus, and mild retraction of the nerve root or dural sac with a UBE retractor adequately exposes the annular defect or maximal protrusion site. In contrast, annular suturing for central herniations may require excessive dural traction, while lateral herniations may necessitate additional facet joint resection, potentially compromising postoperative spinal stability. Therefore, only patients with single-level, low lumbar paracentral herniations without annular calcification and with relatively preserved annular integrity after discectomy were included in this study, thereby minimizing selection bias related to technical feasibility.

Biomechanical testing by Yang Yang et al. (18) demonstrated that the tensile strength of the annulus fibrosus is greater along the longitudinal fiber orientation than in the horizontal direction. Therefore, longitudinal or oblique annulotomy is recommended for contained herniations to minimize biomechanical disruption, and the incision length should preferably be less than 5 mm. The suture position should be preplanned before incision, and incisions should be placed where suturing is technically feasible. The suture depth should be sufficient to achieve full-thickness penetration of the annulus fibrosus, and the suture tension should be adequate to ensure effective closure of the annular defect. Advance the knot away from the nerve root to avoid irritation or mechanical interference with the nerve root. For annular defects near the vertebral body margin, suture-anchor–assisted repair may be considered (20). For large defects, multiple parallel or crossed sutures are recommended. Repeated puncture should be avoided during suturing to minimize additional annular damage. In our surgical practice, the number of sutures was determined according to the length of the annular defect, with an inter-suture spacing of approximately 3–5 mm. Successful repair was defined as complete approximation of the annular fissure, with a residual gap of less than 1 mm between the opposing edges of the defect.

In the present study, two patients in the suture group experienced transient aggravation of lower limb numbness postoperatively, both at the L3/4 level. This may be related to the relatively wider dural sac at L3/4, the presence of a greater proportion of cauda equina fibers compared with lower lumbar levels, reduced neural mobility space, and facet joint convergence at this segment. Without increasing bony resection, excessive traction on the nerve root or dura may occur during suturing. Therefore, posterior discectomy combined with annular suturing at L3/4 or higher levels should be applied with caution. Although annular suturing prolonged the operative time, no obvious time-related complications were observed in our cohort, which may be attributable to the limited increase in operative duration, careful patient selection, and standardized perioperative management. Strict indication control is essential, and decisions regarding annular suturing should be based on comprehensive evaluation of herniation level, zone, calcification status, degeneration grade, defect size and morphology, and the quality of surrounding annular tissue.

This study has several limitations. First, this was a single-center retrospective study with a relatively small sample size and short follow-up duration, which may limit the generalizability of the findings. In addition, invasive evaluation of annular healing and mechanical properties after suturing was not feasible. Therefore, larger prospective studies with standardized defect classification and longer follow-up are needed to further validate our findings.

In this retrospective study of selected patients with single-level lower lumbar paracentral lumbar disc herniation (LDH), both unilateral biportal endoscopic (UBE) discectomy alone and UBE discectomy combined with annular suturing achieved favorable short-term clinical improvement. Although the suture group demonstrated less severe early postoperative leg pain, along with lower recurrence rates and less radiographic progression, these differences did not reach statistical significance between groups. Furthermore, annular suturing was associated with increased operative time. Therefore, the potential added value of annular repair should be interpreted with caution and requires confirmation in larger prospective studies.

## Data Availability

The raw data supporting the conclusions of this article will be made available by the authors, without undue reservation.
